# Lung elastance can be determined without esophageal pressure measurements

**DOI:** 10.1186/cc12068

**Published:** 2013-03-19

**Authors:** C Grivans, S Lundin, O Stenqvist

**Affiliations:** 1 Sahlgrenska University Hospital, Gothenburg, Sweden

## Introduction

We have previously shown, in an *ex vivo *porcine model, that lung elastance calculated as the PEEP change divided by lung volume increase (ΔPEEP/ΔEELV) is closely correlated to conventionally measured lung elastance using oesophageal pressure [1]. In this study we hypothesize that the successive change in lung volume during a PEEP-step manoeuvre could be predicted from ΔPEEP and lung elastance as ΔPEEP/EL. The objective of the study was to validate this hypothesis in patients with acute respiratory failure (ARF).

## Methods

Thirteen patients with ARF were studied during an incremental PEEP trial, 0-4-8-12-16 cmH_2_O. ΔEELV was determined as the change in expiratory tidal volume following each PEEP step. Conventional calculation of lung elastance was obtained from tidal variation in airway pressure minus tidal variation in oesophageal pressure divided by tidal volume. Position of the oesophageal catheter was verified according to Baydur [2]. The measured change in end-expiratory lung volume during the PEEP-step manoeuvre using spirometry was compared with the end-expiratory lung volume change calculated from EL and stepwise changes in PEEP as ΔPEEP/EL.

## Results

**Figure 1:**
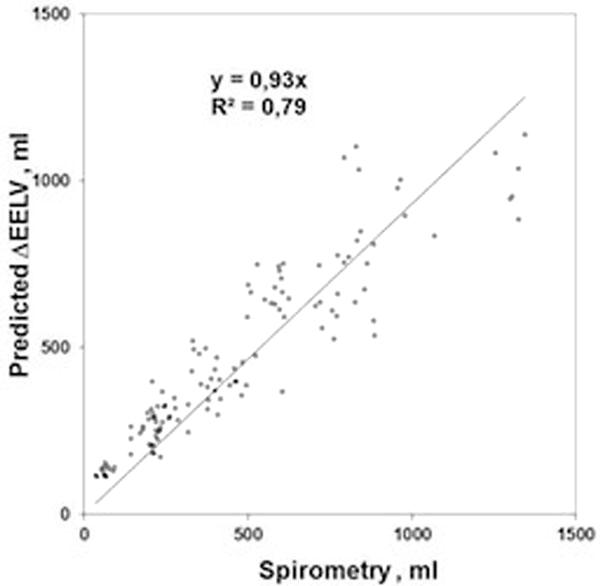
**Correlation between spirometrically measured and calculated increase in EELV**.

There was a close correlation between the measured build-up of end-expiratory lung volume during a PEEP-step manoeuvre and ΔPEEP/EL where EL was conventionally determined using oesophageal pressure measurements (see Figure 1).

## Conclusion

Esophageal pressure measurements are difficult to perform [3] and rarely used in routine clinical practice. Our findings indicate that a change in PEEP together with measurements of the resulting change in end-expiratory volume by spirometry in the ventilator could be used to determine lung elastance separately, the relation between lung and chest wall elastance as well as the transpulmonary pressure.
